# Latent Healthcare Stigma Profiles and Their Association With Human Immunodeficiency Virus (HIV) Treatment and Care Outcomes Among Women With HIV in the United States: An Intersectional Analysis

**DOI:** 10.1093/ofid/ofaf414

**Published:** 2025-08-06

**Authors:** Jennifer P Jain, Nadra E Lisha, Jae Sevelius, Torsten B Neilands, Carol Dawson-Rose, Mallory O Johnson, Ayden Scheim, Bulent Turan, Adebola Adedimeji, Mirjam-Colette Kempf, Gypsyamber D’Souza, Michelle Floris-Moore, Seble Kassaye, Anandi Sheth, Azure Thompson, Deborah Jones Weiss, Phyllis C Tien

**Affiliations:** Department of Community Health Systems, School of Nursing, University of California, San Francisco, San Francisco, California, USA; Division of Prevention Science, School of Medicine, University of California, San Francisco, San Francisco, California, USA; Department of Psychiatry, Columbia University, New York, New York, USA; Division of Prevention Science, School of Medicine, University of California, San Francisco, San Francisco, California, USA; Department of Community Health Systems, School of Nursing, University of California, San Francisco, San Francisco, California, USA; Division of Prevention Science, School of Medicine, University of California, San Francisco, San Francisco, California, USA; School of Public Health, Drexel University, Philadelphia, Pennsylvania, USA; Department of Psychology, Koc University, Istanbul, Turkey; Department of Epidemiology and Population Health, Albert Einstein College, New York, New York, USA; Schools of Nursing, Public Health and Medicine, University of Alabama at Birmingham, Birmingham, Alabama, USA; Bloomberg School of Public Health, Johns Hopkins University, Baltimore, Maryland, USA; Division of Infectious Diseases, University of North Carolina School of Medicine, Chapel Hill, North Carolina, USA; Department of Medicine, Georgetown University, Washington, District of Columbia, USA; Department of Medicine, Emory University, Atlanta, Georgia, USA; Community Health Sciences, State University of New York Downstate Health Sciences University, New York, New York, USA; Department of Psychiatry and Behavioral Sciences, University of Miami, Miami, Florida, USA; Department of Medicine, University of California, San Francisco, San Francisco, California, USA

**Keywords:** HIV, intersectionality, substance use, women

## Abstract

**Background:**

Stigma is a barrier to human immunodeficiency virus (HIV) care among women with HIV (WWH) in the United States (US). We estimated associations between latent stigma profiles and HIV outcomes among WWH in the Women's Interagency HIV Study.

**Methods:**

From 2018 to 2019, participants (N = 1407) completed semi-annual assessments on sociodemographics, substance use, HIV-related, anticipated, and race-related stigma in healthcare, and suboptimal antiretroviral therapy adherence (<95%), and underwent HIV RNA testing. Latent profile analysis and multinomial logistic regression were used to examine adjusted associations between profiles and several covariates. Structural equation modeling estimated longitudinal associations between profiles, suboptimal adherence, and viral nonsuppression (HIV-1 RNA ≥20 copies/mL).

**Results:**

We identified 3 profiles: high stigma (3%), low stigma (86%), and anticipated stigma (11%). Membership in the high stigma profile was greater for Black WWH who use drugs (adjusted odds ratio [aOR], 3.6 [95% confidence interval {CI}, 1.1–12.1]), non-Black WWH who use drugs (aOR, 4.8 [95% CI, 1.3–18]), and those who reported suboptimal adherence (aOR, 2.2 [95% CI, 1–4.8]), drug use (aOR, 2.6 [95% CI, 1.3–5.1]), noninjection drug use (aOR, 2.2 [95% CI, 1.1–4.4]), opioid use treatment (aOR, 4.07 [95% CI, 1.47–11.26]), depression (aOR, 5.8 [95% CI, 2.8–11.9]), stress (aOR, 1.09 [95% CI, 1.05–1.1]), and high post-traumatic stress disorder (aOR, 10.6 [95% CI, 4.3–25.7]). In the longitudinal model, suboptimal adherence was lowest for the low stigma profile and predicted future viral nonsuppression.

**Conclusions:**

Reducing stigma and integrating HIV, substance use, and mental health treatment is crucial for improving health outcomes among US WWH.

In the era of treatment as prevention (TasP), optimal adherence to antiretroviral therapy (ART) is vital to attaining human immunodeficiency virus (HIV) viral suppression and preventing the onward transmission of HIV [1]. Despite advances in HIV treatment, troubling disparities in HIV treatment outcomes persist among women with HIV (WWH) who use drugs and Black WWH in the United States (US) [2–8]. Black WWH are disproportionately affected by structural barriers to HIV care, including limited access to culturally tailored healthcare services and racial discrimination [9]. These barriers may be more intensified among Black WWH who use drugs, because they experience overlapping stigmas related to their HIV status, race, and drug use [10]. These intersecting forms of stigma are known to contribute to lower rates of engagement in HIV care, adherence to ART, and viral suppression. For example, WWH who use drugs have an 87% higher prevalence of suboptimal adherence to ART compared to WWH who do not use drugs [11]. Similarly, Black WWH report significantly lower rates of adherence compared to other ethnoracial groups, and their mortality rate due to HIV is 17 times higher than that of White WWH [6–8].

Intersectionality is a critical theoretical framework that highlights how systems of power and privilege—including sexism, racism, and substance use–related stigma—create and reinforce social inequities, while acknowledging resilience and resistance to stigma [12–14]. Intersectional stigma and discrimination continue to drive inequalities in HIV treatment outcomes among WWH who use drugs and Black WWH through inhibiting optimal engagement in HIV care [15–20]. Intersectional stigma and discrimination are defined as polices, practices, and behaviors that marginalize stigmatized individuals and perpetuate health inequities [12, 21]. Specifically, among WWH who use drugs, racism, sexism, HIV stigma, and substance use stigma often lead to suboptimal ART adherence and viral nonsuppression through an escalation of harmful drug use and healthcare avoidance [10, 15, 22–26]. On the contrary, resilience is a critical resource that may moderate stigma's impact on HIV care among WWH who use drugs and Black WWH; however, its role in this context remains understudied [27–29].

To alleviate the burden of HIV among WWH who use drugs and Black WWH, it is essential to mitigate the impact of intersectional stigma and discrimination and enhance resilience [30]. To do this, research with clear theoretical linkages to intersectionality is needed. Further, when utilizing data from racially diverse cohorts of WWH with varying patterns of drug use, there is a need to identify similar subpopulations based on their endorsement of different stigmas in healthcare settings to understand how distinct stigma profiles differentially predict HIV treatment outcomes across multiple axes of discrimination based on drug use and race. In addition, there is a need to understand how distinct profiles differentially predict inequalities in HIV treatment outcomes over time using longitudinal models [31].

To address these gaps in research, we used a latent profile analysis (LPA) in conjunction with a longitudinal path model as recommended by prominent experts focused on intersectionality in quantitative research [32, 33]. These methods were applied to evaluate how distinct stigma profiles differentially predict suboptimal adherence, and then how suboptimal adherence predicts viral nonsuppression across intersections of drug use and race among US WWH. This person-centered approach evaluates intersectionality by treating different patterns of experienced stigmas as latent empirically determined constructs, while incorporating a social grouping predictor variable that spans multiple axes of discrimination [33]. We hypothesized that profiles characterized by higher levels of stigma would be associated with suboptimal adherence and viral nonsuppression, and that WWH who use drugs and Black WWH who use drugs will be more likely to be in profiles defined by higher levels of stigma. For the longitudinal component, we hypothesized that profiles defined by stigma would predict suboptimal adherence and suboptimal adherence would in turn predict future viral nonsuppression. As such, this research may shed light on how oppression at the intersections of drug use and race among US WWH, stigma, and HIV care outcomes interact, offering insights to inform public health programs and policies that promote intersectional equity and TasP among WWH [34–37].

## METHODS

### Study Population

The Women's Interagency HIV Study (WIHS) established in 1994, is the oldest and largest ongoing prospective cohort study of women with HIV and sociodemographically similar women without HIV in the US [38]. It is now integrated with the Multicenter AIDS Cohort Study (MACS) and has become the MACS/WIHS Combined Cohort Study (MWCCS). Women are recruited into the MWCCS through several outreach strategies conducted at clinical sites and community settings across the US. MWCCS enrolls WWH as well as demographically similar women without HIV. Participants are identified through HIV clinics, community-based organizations, and word-of-mouth referrals. The cohort is designed to reflect the demographic profiles of women affected by HIV across the US, with extra effort placed on recruiting underserved women.

This study leveraged WIHS data collected among women with HIV only; women without HIV were excluded from the present study. Data collected between 1 April 2018 and 30 September 2019 were utilized for this study. Data from the following sites were leveraged for the present study/analysis: the Bronx and Brooklyn, New York; Chicago, Illinois; San Francisco, California; Los Angeles, California; Washington, District of Columbia; Atlanta, Georgia; Chapel Hill, North Carolina; Miami, Florida; Jackson, Mississippi; and Birmingham, Alabama.

### Study Procedures

WIHS procedures and protocols are described fully elsewhere [38]. In brief, data on sociodemographics, substance use, stigma, mental health, and HIV treatment and care were collected semiannually, via an interview-administered questionnaire. Women underwent HIV viral load testing every 6 months. For this analysis, one's baseline visit was considered the first visit on record during the study period, which includes 3 visits (visits 48, 49, and 50). Participants were not required to complete all 3 study visits (visits 48–50) to be included in the analysis. Participants provided written informed consent, and study activities took place in a private setting. Study procedures were approved by the institutional review boards at each site.

### Measures

#### Experienced Stigmas in Healthcare Settings

**Figure 1.**
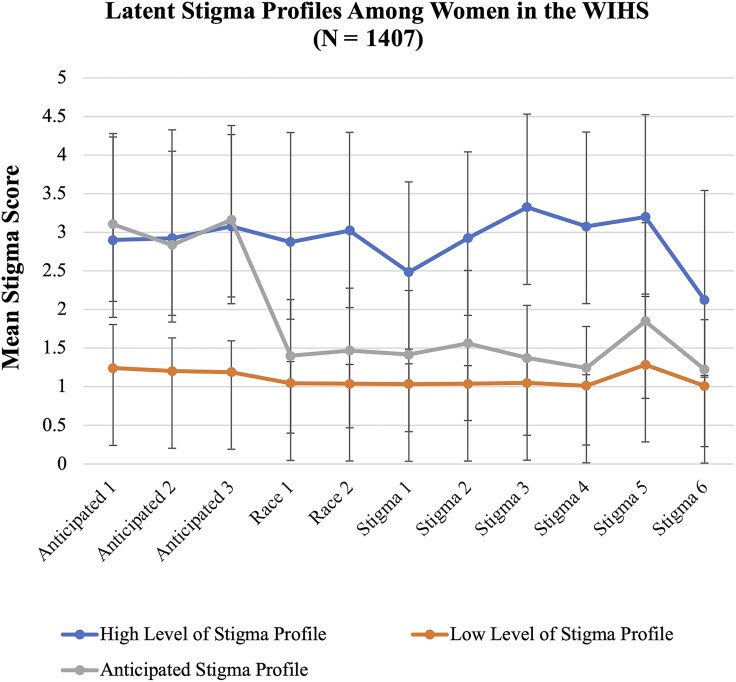
This figure illustrates the separation between each latent profile and homogeneity within each profile. The mean stigma score for each type of stigma is plotted, along with standard errors to show the variability around each mean score. Here are the items for each stigma measure: Anticipated HIV Stigma in Healthcare Setting, 1–3: (1) “Healthcare workers will not listen to my concerns”; (2) “Healthcare workers will avoid touching me”; and (3) “Healthcare workers will treat me with less respect.” Experienced Race-Related Stigma in Healthcare Settings, 1–2: (1) “In the past 12 months, how often did healthcare workers pay less attention to you because of your race or ethnicity?” and (2) “In the past 12 months, how often did you feel discriminated against by healthcare workers because of your race or ethnicity?” Experienced HIV Stigma in Healthcare Settings, 1–6: (1) “Healthcare workers were unwilling to care for you because you are living with HIV”; (2) “Healthcare workers provided poorer quality of care to you than to other patients because you are living with HIV”; (3) “Healthcare workers talked badly about people living with HIV”; (4) “Healthcare workers disclosed or told your HIV status to others without your permission”; (5) “Healthcare workers used extra infection control precautions (like wearing extra gloves) when caring for you because you are a person living with HIV”; and (6) “Healthcare workers sent or referred you to another health facility because the workers do not want to treat you there.” Abbreviations: HIV, human immunodeficiency virus; WIHS, Women's Interagency HIV Study.

We used validated measures of experienced HIV-related stigma, race-related stigma, and anticipated stigma in healthcare settings at visit 48. We assessed anticipated HIV-related stigma using a 3-item measure and subscale from the HIV Stigma Framework Scale [39] (eg, “Healthcare workers will not listen to my concerns”) and a Likert response scale (1 = very unlikely to 5 = very likely); the Cronbach coefficient was α = .89. We assessed experienced race-related stigma or implicit racial bias in healthcare settings using a 2-item measure from the discrimination subscale of the Interpersonal Processes of Care Survey [40] (eg, “In the past 12 months, how often did you feel discriminated against by healthcare workers because of your race or ethnicity?”) and a Likert scale (1 = never to 5 = almost always); the Cronbach coefficient was α = .86. We measured experienced HIV-related stigma using a 6-item measure adapted from the experienced stigma subscale measuring HIV-related stigma among health facility staff [41] (eg, “Healthcare workers disclosed or told your HIV status to others without your permission”) and the same Likert scale; the Cronbach coefficient was α = .81. All stigma items are displayed in Figure 1.

#### Sociodemographics

Sociodemographic factors utilized for this study include employed (yes/no), education (high school or more vs less than high school), age in years, average annual income ( >$24 000 vs ≤$24 000), married (yes/no), and unstably housed (yes/no).

#### Intersectional Groupings

Data on race and drug use were used to create 4 intersectional groupings as predictors of profile membership. A dichotomous measure of any drug use (yes/no) was created by placing those who reported using any of the following drugs in the past 6 months in the drug use category: cannabis, methamphetamine, cocaine, crack cocaine, heroin, nonprescribed prescription opioids, ecstasy, phencyclidine or angel dust, psychedelics, poppers (alkyl nitrites), and nonprescribed benzodiazepines or barbiturates. A dichotomous measure of race, Black versus non-Black, was created to avoid data sparsity issues, as the majority (75%) identified as Black. From these variables, a categorical variable with 4 intersectional groupings was created: (1) non-Black women who do not use drugs (reference group); (2) non-Black women who use drugs; (3) Black women who do not use drugs; and (4) Black women who use drugs.

#### HIV Treatment/Care

Adherence to ART was measured by asking participants to report how often they took their HIV medications as prescribed in the past 6 months (100% of the time; 95%–99% of the time; 75%–94% of the time; <75% of the time). Based on these responses, we created a dichotomous measure of suboptimal adherence to ART (yes/no), defined as reporting <95% [42–44]. In addition, a dichotomous measure of viral nonsuppression (yes/no), was created using a cutoff of HIV-1 RNA ≥20 copies/mL, as viral suppression is defined as levels below the lower limit of detection of the HIV RNA assay, which is 20 copies/mL in the MWCCS. Viral load measurement corresponds to the time of each study visit.

#### Substance Use

As noted above, we created a variable for any drug use in the past 6 months. We also collected data on any noninjection drug use (yes/no); heavy drinking (yes/no), defined as consuming >7 drinks per week; and enrollment in opioid treatment (yes/no), in the past 6 months.

#### Mental Health

Using the Center for Epidemiological Studies Depression Scale [45] and a cutoff of 16, we created a binary variable for being at risk of clinical depression (yes/no). We measured stress using the Perceived Stress Scale–10 [46]; scores range from 0 to 40, and higher scores indicate higher levels of stress. Trust in one's HIV care provider was assessed using the Safran Physician Trust Subscale [47]. Resilience was measured using the Conner-Davidson Resilience Scale [48], where participants responded to 10 statements about personal resilience using a Likert scale (1 = not true at all to 5 = true nearly all the time). All of these variables were collected across the entire study period. PTSD symptoms were assessed using the 17-item Civilian PTSD Checklist (PCL-C), which is designed for the general population. Participants were asked to rate how bothered they were by each symptom in the past month using a 5-point Likert scale ranging from 1 (“Not at all”) to 5 (“Extremely”). Total scores range from 17 to 85, with higher scores indicating higher levels of PTSD symptom severity. Scores were categorized into four severity levels: little or no PTSD symptoms: 17–27, some PTSD symptoms: 28–29, moderate PTSD symptoms: 30–44 and high PTSD symptoms: 45–85 [49].

### Statistical Analyses

#### Latent Profile Analysis

We conducted a LPA using cross-sectional data from visit 48 to identify similar subgroups of women based on their endorsement of (1) experienced HIV-related stigma in healthcare settings; (2) experienced race-related stigma in healthcare settings; and (3) anticipated stigma in healthcare settings. LPA identifies unobserved subgroups by examining observed response patterns from continuous indicators. Since there were only 3 subscales, rather than using summary scores, we utilized individual items from each stigma subscale for the LPA. We fit between 1 and 6 LPA models and selected the 3-profile model as the final solution based on fit indices including Bayesian information criterion (BIC), bootstrapped likelihood ratio test, Vuong-Lo-Mendell-Rubin likelihood ratio test (VLMRT), and entropy, as well as practical considerations such as profile size and the interpretability or the meaningfulness of the profiles (Table 1) [50–52]. Although BIC values continued to decrease with additional classes, improvement plateaued after the 3-class model (BIC, 22 777.2 vs 24 667.0 for the 2-class model). The 3-class solution showed acceptable entropy (0.983), indicating strong classification certainty. Although the VLMRT *P* value was not significant (*P* = .1966), models with 4 or more classes produced small, potentially unstable groups (eg, only 9 individuals in 1 class), raising concerns about overfitting. Thus, we selected the 3-class model as the most parsimonious and conceptually meaningful solution. Participants were assigned to their most likely latent class based on posterior probabilities derived from the LPA. Although this approach does not explicitly account for classification uncertainty, the model entropy was high (1.00), indicating strong class separation and high confidence in class assignment. While not incorporating uncertainty in class membership can lead to underestimated standard errors in downstream analyses, the high entropy and clear profile distinctions support the appropriateness of this approach for the current study. Figure 1 illustrates the separation between each profile and homogeneity within each profile.

**Table 1.** Model Fit Indices for the Latent Profile Analysis With 1–6 Profiles Using 11 Single Items (N = 1407)

Classes (Items)	N1	N2	N3	N4	N5	N6	BIC	BLRT	VLMRT	Entropy
1	1407	…	…	…	…	…	29 451.66	…	…	…
2	1318	89	…	…	…	…	24 667.02	0.00	0.0987	0.994
3	40	1207	160	…	…	…	22 777.17	0.00	0.1966	0.983
4	25	100	1200	82	…	…	21 641.24	0.00	0.6132	0.99
5	44	9	53	1207	94	…	19 974.56	0.00	0.2443	0.992
6	23	27	1196	44	105	12	17 957.08	0.00	0.7382	0.993

Single items, instead of summary scores, were used to fit the latent profile analysis, because only 3 scales were used.

Abbreviations: BIC, Bayesian information criterion; BLRT, bootstrapped likelihood ratio test; N, number of participants in each latent profile; VLMRT, Vuong-Lo-Mendell-Rubin likelihood ratio test.

#### Descriptive Analysis

We analyzed the distribution of several covariates across the 3 profiles to identify those that varied significantly. The χ^2^ test was used for categorical and binary variables, while *t* test was employed for continuous variables, with a significance threshold of *P* < .05 (Table 2). Covariates that showed significant variation across profiles were subsequently included in the multinomial regression analysis to explore the relationship between profile membership and these covariates (Table 3). The covariates of interest included intersectional groupings based on race and drug use, suboptimal adherence and viral nonsuppression, substance use, substance use treatment, sex work, depression, stress, posttraumatic stress disorder (PTSD), and resilience.

**Table 2.** Sociodemographic Characteristics, Intersectional Positions, Substance Use, Mental Health, and Human Immunodeficiency Virus (HIV) Treatment and Care Indicators by Latent Profile Among Women With HIV in the United States (N = 1407)

Variable	Overall (N = 1407)	Profile 1: High Stigma (n = 40 [3%])	Profile 2: Low Stigma (Reference Profile) (n = 1207 [86%])	Profile 3: Anticipated Stigma (n = 160 [11%])	*P* Value: Profile 1 vs 2	*P* Value: Profile 3 vs 2
Intersectional groupings based on drug use and race^a^					.01	.13
Non-Black women who do not use drugs	249 (17.7%)	4 (10.0%)	219 (18.1%)	26 (16.3%)		
Non-Black women who use drugs	96 (6.8%)	6 (15.0%)	74 (6.1%)	16 (10.0%)		
Black women who do not use drugs	775 (55.1%)	17 (42.5%)	678 (56.2%)	80 (50.0%)		
Black women who use drugs	287 (20.4%)	13 (32.5%)	236 (19.5%)	38 (23.8%)		
Sociodemographic factors						
Age, y, mean (SE)	38.68 (9.3)	38.70 (1.5)	38.89 (0.27)	37.08 (0.73)	.99	.05
Average annual income					.76	.10
≤$24 000 on average annually	980 (72.9%)	29 (74.4%)	838 (72.1%)	123 (78.3%)		
>$24 000 on average annually	364 (27.1%)	10 (25.6%)	320 (27.9%)	34 (21.7%)		
Married					.39	.66
No	1093 (79.7%)	29 (74.4%)	940 (80.0%)	124 (78.5%)		
Yes	279 (20.3%)	10 (25.6%)	235 (20.0%)	34 (21.5%)		
Employed					.02	.01
No	883 (63.8%)	32 (80.0%)	736 (61.0%)	115 (71.8%)		
Yes	524 (37.2%)	8 (20.0%)	471 (39.0%)	45 (28.1%)		
Education					.62	.21
Less than high school	450 (32.0%)	14 (35.0%)	378 (31.3%)	58 (36.2%)		
High school or more	956 (68.0%)	26 (65.0%)	828 (68.7%)	102 (63.8%)		
Unstably housed^b^					.43	.70
No	1252 (89.0%)	34 (85.0%)	1074 (89.0%)	144 (90.0%)		
Yes	155 (11.0%)	6 (15.0%)	133 (11.1%)	16 (10.0%)		
Race/Ethnicity						
White, non-Hispanic	139 (9.9%)	4 (10.0%)	119 (9.8%)	16 (10.0%)	.14	.05
White, Hispanic	65 (4.6%)	1 (2.5%)	60 (5.0%)	4 (2.5%)		
Black/African American, non-Hispanic	1035 (73.6%)	30 (75.0%)	890 (73.7%)	115 (71.9%)		
Black/African American, Hispanic	27 (1.9%)	0 (0.0%)	24 (2.0%)	3 (1.9%)		
Other, Hispanic	98 (7.0%)	2 (5.0%)	84 (7.0%)	12 (7.5%)		
Asian/Pacific Islander	6 (0.9%)	0 (0.0%)	3 (0.2%)	3 (1.9%)		
Native American/Native Alaskan	13 (0.9%)	2 (5.0%)	8 (0.7%)	3 (1.9%)		
Other	24 (1.7%)	1 (2.5%)	19 (1.6%)	4 (2.5%)		
HIV treatment and care in the past 6 mo						
Suboptimal adherence to ART^c^					.04	.35
No	1119 (84.8%)	27 (73.0%)	969 (85.5%)	123 (82.5%)		
Yes	201 (15.2%)	10 (27.0%)	165 (14.5%)	26 (17.5%)		
Virally nonsuppressed^d^					.44	.88
No	967 (71.3%)	25 (65.8%)	832 (71.6%)	110 (70.1%)		
Yes	389 (28.7%)	13 (34.2%)	331 (28.4%)	45 (29.0%)		
Time on ART	7.89 (5.2)	9.24 (0.82)	7.76 (0.15)	8.51 (0.41)	.18	.20
Substance use and related factors in the past 6 mo						
Heavy drinking^e^					.76	.38
No	1313 (93.4%)	37 (92.5%)	1129 (93.7%)	147 (91.9%)		
Yes	92 (6.6%)	3 (7.5%)	76 (6.3%)	13 (8.1%)		
Any drug use^f^					.00	.03
No	1024 (72.8%)	21 (52.5%)	897 (74.3%)	106 (66.2%)		
Yes	383 (27.2%)	19 (47.5%)	310 (25.7%)	54 (22.8%)		
Marijuana use					.02	.03
No	1106 (78.7%)	26 (65.0%)	964 (80.0%)	116 (72.5%)		
Yes	299 (21.3%)	14 (35.0%)	241 (20.0%)	44 (27.5%)		
Crack cocaine use					.39	.20
No	1337 (95.1%)	37 (92.5%)	1151 (95.4%)	149 (93.1%)		
Yes	69 (4.9%)	3 (7.5%)	55 (4.6%)	11 (6.9%)		
Cocaine use					.56	.90
No	1359 (96.7%)	38 (95.0%)	1166 (96.7%)	155 (96.9%)		
Yes	47 (3.3%)	2 (5.0%)	40 (3.3%)	5 (3.1%)		
Heroin use					.14	.33
No	1396 (99.3%)	39 (97.5%)	1199 (99.4%)	158 (98.8%)		
Yes	47 (3.3%)	1 (2.5%)	7 (0.6%)	2 (1.2%)		
Noninjection drug use					.01	.00
No	1058 (75.3%)	24 (60.0%)	925 (76.7%)	109 (68.1%)		
Yes	348 (24.7%)	16 (40.0%)	281 (23.3%)	51 (31.9%)		
Enrolled in an opioid use treatment program					.00	.66
No	1360 (96.7%)	35 (87.5%)	1169 (96.9%)	156 (97.5%)		
Yes	47 (3.3%)	5 (12.5%)	38 (3.1%)	4 (2.5%)		
Ever engaged in sex work^g^					.40	.55
No	934 (66.5%)	24 (60.0%)	800 (66.4%)	110 (68.8%)		
Yes	471 (33.5%)	16 (40.0%)	405 (33.6%)	50 (31.2%)		
Mental health/psychosocial factors in the past 6 mo						
At risk of clinical depression^h^					<.0001	<.0001
No	957 (68.2%)	12 (31.6%)	865 (71.8%)	80 (50.0%)		
Yes	446 (31.8%)	26 (68.4%)	340 (28.2%)	80 (50.0%)		
PTSD					<.0001	.00
Little or no PTSD symptoms (score 17–27)	763 (56.2%)	9 (22.5%)	679 (58.6%)	75 (47.2%)		
Some PTSD symptoms (score 28–29)	74 (5.4%)	3 (7.5%)	65 (5.6%)	6 (3.8%)		
Moderate PTSD symptoms (score 30–44)	308 (22.8%)	10 (25.0%)	260 (22.4%)	38 (23.9%)		
High PTSD symptoms (score 45–85)	212 (15.6%)	18 (45.0%)	154 (13.4%)	40 (25.2%)		
Perceived stress score^i^, mean (SE)	16.84 (7.49)	21.22 (1.18)	16.46 (0.22)	18.49 (0.59)	.00	.00
Trust in HIV care providers score^j^, mean (SE)	3.44 (0.41)	3.42 (0.81)	3.46 (0.93)	3.39 (0.13)	.81	.13
Resilience score^k^, mean (SE)	4.03 (0.73)	3.77 (0.12)	4.06 (0.02)	3.82 (0.06)	.03	.00

Data are presented as No. (%) unless otherwise indicated. These data were drawn from the baseline visit for this study (visit 48). Participants were asked to report on their engagement in these behaviors since their last study visit, and study visits occurred every 6 months. PTSD symptoms were assessed using the 17-item Civilian PTSD Checklist (PCL-C) [53], which is designed for the general population. Participants were asked to rate how bothered they were by each symptom in the past month using a 5-point Likert scale ranging from 1 (“Not at all”) to 5 (“Extremely”). Total scores range from 17 to 85, with higher scores indicating higher levels of PTSD symptom severity. Scores were categorized into four severity levels: little or no PTSD symptoms: 17–27, some PTSD symptoms: 28–29, moderate PTSD symptoms: 30–44 and high PTSD symptoms: 45–85.

Abbreviations: ART, antiretroviral therapy; HIV, human immunodeficiency virus; PTSD, posttraumatic stress disorder; SE, standard error.

^a^Intersectional identities and practices were based on self-reported race and any drug use since one's last visit.

^b^Individuals were classified as unstably housed if they reported living in someone else's house, a halfway house, a shelter/welfare hotel, on the street, in a drug treatment facility, or any “other” location.

^c^Suboptimal adherence to ART was defined as <95% self-reported adherence in the past 6 months.

^d^HIV viral load was collected every 6 months and detectable viral load was defined as a viral load of ≥20 copies/mL.

^e^Heavy drinking was defined as consuming >7 drinks per week in the past 6 months.

^f^Any drug use combined any illicit recreational use and marijuana use in the past 6 months.

^g^A history of sex work included women who reported ever having sex for drugs, money, or shelter.

^h^Participants were classified as at risk of clinical depression if they received a score of ≥16 on the Center for Epidemiologic Studies Depression Scale.

^i^Stress was measured using the Perceived Stress Scale–10, where scores range from 0 to 40 and higher scores represent higher levels of stress.

^j^Trust in HIV care providers scores range from 1 to 5, with lower scores representing lower levels of trust and higher scores representing higher levels of trust in HIV care providers.

^k^Resilience was measured using the Brief Resilience Scale; scores range from 1 to 5, and higher scores represent higher levels of resilience.

**Table 3.** Adjusted Associations Between Sociodemographic Characteristics, Intersectional Positions, Substance Use, Mental Health, and Human Immunodeficiency Virus (HIV) Treatment Care Indicators and Profile Membership Among Women With HIV in the United States (N = 1407)

Variable	Profile 1: “High Stigma”	*P* Value	Profile 3: “Anticipated Stigma”	*P* Value
Intersectional groupings				
Black women who do not use drugs	1.70 (.55–5.20)	.36	1.12 (.69–1.82)	.66
Black women who use drugs	3.67 (1.12–12.10)	.03	1.45 (.83–2.54)	.19
Non-Black women who use drugs	4.88 (1.32–18.00)	.02	1.75 (.87–3.52)	.11
Non-Black women who do not use drugs (reference class)	…		…	
Suboptimal adherence to ART	2.29 (1.08–4.86)	.04	1.24 (.78–1.96)	.37
Any drug use	2.65 (1.38–5.11)	.003	1.40 (.97–2.01)	.07
Marijuana use	2.16 (1.08–4.32)	.03	1.44 (.98–2.11)	.06
Noninjection drug use	2.26 (1.15–4.42)	.02	1.49 (1.03–2.15)	.03
Enrolled in opioid use treatment	4.07 (1.47–11.26)	.006	0.69 (.24–1.97)	.49
At risk of clinical depression	5.84 (2.85–11.97)	<.0001	2.58 (1.82–3.65)	<.0001
Employed	0.36 (.15–.87)	.02	0.60 (.39–.91)	.02
Resilience score	0.59 (.39–.91)	.01	0.65 (.52–.81)	.0002
Perceived stress score	1.09 (1.05–1.14)	<.0001	1.04 (1.01–1.06)	.003
High PTSD	10.63 (4.38–25.79)	<.0001	2.43 (1.56–3.78)	<.0001
Moderate PTSD	3.07 (1.22–7.74)	.02	1.33 (.87–2.05)	.19
Some PTSD	4.41 (1.14–17.06)	.03	0.90 (.37–2.17)	.81
Little or no PTSD (reference class)	…		…	

Data are presented as adjusted odds ratio (95% confidence interval) unless otherwise indicated.

All models controlled for age in years, time on ART, education, income, and marital status. Data were collected semi-annually, and respondents were asked to report on their engagement in certain risk behaviors since their last visit. Adjusted associations of profile membership are in comparison to membership in profile 2, the “low stigma” profile.

Abbreviations: ART, antiretroviral therapy; PTSD, posttraumatic stress disorder.

#### Multinomial Logistic Regression

To examine the association between profile membership and the covariates of interest, we built a series of multinomial logistic regression models. Each covariate was modeled separately, and we report the total effects of each exposure on the outcome. All models were adjusted for age in years, time on ART, education, income, and marital status. These variables were selected based on prior MWCCS research identifying them as correlates of HIV treatment and care outcomes, and were conceptualized as confounders in our analysis [54]. Analyses were completed using SAS version 9.4 software.

#### Longitudinal Analysis

To measure changes over time and account for temporal ordering, we leveraged the longitudinal data structure and analyzed how distinct stigma profiles at visit 48 differentially predicted suboptimal adherence at visit 49, and how suboptimal adherence predicted viral nonsuppression at visit 50, using a structural equation model. Specifically, we used significant predictors (*P* < .05) from the multinomial regression results (at visit 48) to predict latent stigma profile membership at visit 48, and then used latent stigma profile membership to predict suboptimal adherence at visit 49 to see if latent stigma profile membership impacted suboptimal adherence. Finally, the model assessed how suboptimal adherence at visit 49 predicted viral nonsuppression at visit 50. These models controlled for the same confounders listed above. To address missing data and provide more robust and reliable estimates, multiple imputation was performed using Bayesian estimation with an unrestricted model [55]. Analyses were performed using M*plus* version 8.11.

## RESULTS

A total of 1407 WWH were included in this analysis, of whom 10% were non-Hispanic White, 5% were Hispanic White, 73% were non-Hispanic Black, 2% were Hispanic Black, 7% were other Hispanic, 0.4% were Asian/Pacific Islander, 0.9% were Native American/Native Alaskan, and 1.7% identified as another race/ethnicity. Regarding intersectional groupings, 55% were Black women who do not use drugs, 20% were Black women who use drugs, 18% were non-Black women who do not use drugs, and 7% were non-Black who use drugs. The mean age was 38.7 years (standard deviation [SD], 9.3 years), the majority (72%) reported earning ≤$24 000 on average annually, most (79%) were not married, many were unemployed (63%), and 68% completed high school or more. Everyone reported a history of incarceration, 33% reported a history of sex work, and nearly all (89%) reported being stably housed. Only 15% reported suboptimal adherence to ART, 28% were virally unsuppressed, and the mean time on ART was 7.9 years (SD, 5.2 years). At visit 48, 28.73% of participants were virally nonsuppressed; this increased slightly to 29.12% at visit 49 and declined to 25.94% at visit 50. Transitions in viral suppression status between visits revealed that some participants moved from viral suppression to viral nonsuppression and vice versa, indicating fluctuation in HIV treatment outcomes over time.

Few women (6%) reported heavy drinking, over a quarter (27%) reported any drug use, 24% reported any noninjection drug use, 21% reported cannabis use, 4% reported crack cocaine use, 3% reported cocaine use, 3% reported heroin use, and very few (3%) reported being enrolled in an opioid treatment program. Many (30%) were at risk of clinical depression, the majority reported few or no PTSD symptoms (56%), followed by moderate symptoms (22%), high symptoms of PTSD (15%), and some symptoms (5%). The mean perceived stress score was 16 (standard error [SE], 7), indicating a moderate level of stress overall; the mean trust in HIV care providers score was 3 (SE, 0.4) indicating a moderate level of trust overall; and the mean resilience score was 4 (SE, 0.7), indicating an average level of resilience in relation to population norms.

### Latent Profile Analysis

We identified 3 profiles of WWH based on their endorsement of experienced HIV-related stigma, race-related stigma, and anticipated stigma in healthcare settings. Profile 1 was characterized by a high level of stigma in healthcare settings overall (3%), profile 2 was characterized by a low level of stigma in healthcare settings overall (86%), and profile 3 was characterized by anticipated stigma in healthcare settings (11%).

Compared to the low stigma profile, a significantly greater proportion of women in the high stigma profile reported being Black WWH who use drugs (32% vs 19%) or non-Black WWH who use drugs (15% vs 6%), any drug use (47% vs 25%), suboptimal ART adherence (27% vs 14%), and enrollment in an opioid treatment program (12% vs 3%). Compared to the low stigma profile, a significantly greater proportion of women in the high stigma and anticipated stigma profiles reported being unemployed (80% vs 61% and 71% vs 61%), cannabis use (35% vs 20% and 27% vs 20%), noninjection drug use (40% vs 23% and 31% vs 23%), clinical depression (68% vs 28% and 50% vs 28%), and higher mean (SE) stress scores (21 [1.18] vs 16 [0.22] and 18 [0.59] vs 16 [0.22]). Finally, women in the low stigma profile had significantly higher levels of resilience compared to those in the high stigma profile and the anticipated stigma profile (4.06 [0.02] vs 3.77 [0.12] and 4.06 [0.02] vs 3.82 [0.06]) (Table 2).

Compared to the low stigma profile, the odds of membership in the high stigma profile were higher for Black WWH who use drugs, non-Black WWH who use drugs, and those who reported suboptimal adherence, any drug use, cannabis use, noninjection drug use, enrollment in a opioid treatment program, depression, higher stress levels, and any PTSD. Compared to membership in the low stigma profile, the odds of membership in the anticipated stigma profile were higher for those who reported; noninjection drug use, depression, higher levels of stress, and high PTSD. Women who were employed had a significantly lower odds of membership in the high stigma profile and the anticipated stigma profile compared to the low level of stigma profile. Similarly, those who reported higher levels of resilience had a significantly lower odds of membership in the high stigma profile and the anticipated stigma profile compared to the low level of stigma profile (Table 3).

### Longitudinal Analysis Results

In the longitudinal model, which controlled for age, time on ART, education, income, and marital status, we found that depression (at baseline) was related to being in the anticipated stigma profile (adjusted odds ratio [aOR], 2.00 [95% confidence interval {CI}, 1.30–3.09]) and the high stigma profile (aOR, 2.94 [95% CI, 1.25–6.95]) versus the lower stigma profile. High PTSD (at baseline) (odds ratio [OR], 5.47 [95% CI, 1.37–21.83]) was related to being in the high level of stigma profile compared to the low level of stigma profile. Profile membership was related to suboptimal ART adherence, such that suboptimal adherence was lowest for those in the low level of stigma profile (profile 1 vs 2: OR, 0.46 [95% CI, .22–.96]; profile 3 vs 2: OR, 0.62 [95% CI, .41–.94]). Finally, suboptimal adherence at timepoint 2 predicted future viral nonsuppression at timepoint 3 (OR, 0.31 [95% CI, .22–.42]).

## DISCUSSION

We identified several important findings in this large intersectional study examining subgroups of US WWH based on their endorsement of experienced stigmas in healthcare settings, and their association with suboptimal adherence and viral nonsuppression. While most women in our study reported a low level of stigma in healthcare settings overall, we did identify 2 socially and structurally marginalized groups of women defined by high levels of stigma and anticipated stigma. Our longitudinal path model revealed that these higher stigma profiles predicted suboptimal adherence, which in turn predicted viral nonsuppression. As such, we used longitudinal modeling to estimate the temporal relationships between stigma profiles, ART adherence, and viral nonsuppression, and moved beyond cross-sectional associations to model how stigma in healthcare settings contributes to downstream HIV treatment and care outcomes. Altogether, these findings may help inform the development of multilevel interventions to optimize HIV treatment and intervention among US WWH who use drugs.

The high stigma profile was characterized by women who reported elevated levels of HIV-related, race-related, and anticipated stigma in healthcare settings. This profile had a higher prevalence of Black WWH who use drugs and non-Black WWH who use drugs. Additionally, women in this group were more likely to report drug use, opioid use treatment, suboptimal adherence to ART, depression, unemployment, higher levels of stress, PTSD, and lower levels of resilience, compared to women in the low stigma profile. Similarly, women who reported the following were more likely to be in the anticipated stigma profile: any noninjection drug use, poor mental health conditions, unemployment, and lower levels of resilience. These anticipated findings point to a clear relationship between different stigmas in healthcare settings and women who are multiply marginalized for drug use, poor adherence, psychological morbidity, and economic marginalization. Hence these findings highlight the need to combat stigma and deliver programs that integrate trauma-informed HIV care, substance use treatment, mental health support, and economic empowerment strategies among US WWH [56, 57]. Additionally, it is important to involve WWH with lived experience of stigma in healthcare settings in the design and implementation of such programs and to acknowledge how interlocking systems of power and privilege shape the experiences of WWH [37, 58–60].

Our finding that profiles defined by higher levels of stigma in healthcare settings predicted future suboptimal adherence, which in turn predicted future viral nonsuppression, was consistent with our main hypothesis, which predicted that women with higher levels of stigma would have worse HIV treatment outcomes. This model demonstrates that suboptimal adherence to ART mediates the relationship between stigma and viral nonsuppression, emphasizing that stigma is an important modifiable upstream factor in improving adherence, and ultimately, viral suppression among women. Furthermore, although some of the anticipated relationships in the longitudinal analysis were no longer significant—likely due to the inclusion of other variables that account for shared variance, thereby reducing the unique contribution of the initial predictor—depression and PTSD remained key factors in shaping the higher-risk groups' HIV outcomes. This highlights how interconnected forms of stigma are associated with psychological morbidity and impact HIV care. Altogether, these results suggest the need to challenge social power structures and systems that perpetuate these inequalities among US WWH, through progressive policy reform, and expanded access to integrated care models that comprehensively address HIV, mental health (eg, depression and trauma), and other relevant factors, including substance use and economic empowerment [37, 61, 62].

Integrated care models that address both HIV and substance use are increasingly recognized as essential to improve the health of people with HIV who use drugs. Innovative strategies including mobile health clinics, telehealth platforms, and peer-led outreach that reduce structural barriers, including stigma, transportation challenges, and disjointed service delivery, have been shown to improve health outcomes among individuals affected by both HIV and substance use. For example, low-barrier models that combine HIV care, substance use treatment, and mobile pharmacy access through community health workers have shown promise in improving health outcomes among people who are dually impacted by HIV and drug use [63]. In addition, recent research highlights the value of person-centered, human rights-based approaches that address the underlying social, economic, and political inequalities that drive health inequities among this population [64]. Altogether, there is growing consensus that integrated models grounded in shared decision-making, trauma-informed care, cultural responsiveness, and human rights, all of which align with the principles of harm reduction, are critical for sustaining long-term engagement in HIV care and substance use treatment. Our research contributes to this literature by underscoring the importance of integrated programs that specifically focus on WWH who use drugs that address the co-occurring challenges related to stigma, trauma, mental health, substance use, HIV, and economic marginalization.

This study has several limitations that should be considered. The data utilized for this study were collected between 1 April 2018 and 30 September 2019 and therefore may not be reflective of current trends among WWH in the US. Future studies are planned to utilize data collected more recently to explore the role of intersectional stigma and discrimination on inequalities in HIV treatment and care outcomes among racially diverse WWH in the US. Cross-sectional data were used to create the LPA, and therefore temporal relationships are unclear and causality cannot be determined. Although we controlled for several known correlates of HIV treatment and care outcomes among US women, it is possible that residual or unmeasured confounding affected our results. Latent variable approaches make several assumptions, including unobserved heterogeneity and local independence, and the accuracy of these methods can be affected by data sparsity. Given the small percentage of women in the high stigma profile, whether reflecting a true finding or potential underreporting, we recommend that future studies with larger samples of WWH be conducted to further assess experiences of healthcare stigma.

We acknowledge that this study did not analyze measures of social inequality or power, which are core constructs of intersectionality; therefore, our findings provide an incomplete understanding of how these factors shape the experiences of WWH. Similarly, substance use stigma was not measured; thus, our findings do not capture how this dimension of stigma relates to the profiles identified or the health outcomes assessed. Moreover, non-Black participants, including those of Hispanic ethnicity, may also experience discrimination, and these forms of stigma are important but could not be meaningfully disaggregated in our analysis due to sample size limitations. Nonprobability recruitment methods were used to recruit women into the WIHS, which affects the generalizability of our results to other populations of WWH in the US. Further, because women in the WIHS have regular access to HIV treatment and care and are being retained in a longitudinal cohort study, they may not be reflective of other WWH. We relied upon self-reported data of several sensitive variables including stigma, substance use, and adherence to ART, which are subject to social desirability bias. Due to small cell sizes for some combinations of variables, the CIs for some estimates are very wide.

## CONCLUSIONS

Our study utilized a latent variable person-centered approach to characterize experiences of stigma in healthcare settings among a large, racially diverse cohort of US WWH. While prior research has examined individual forms of stigma in isolation, we used a latent profile analysis to understand how stigma experiences cluster and impact HIV treatment and care outcomes over time among women. Findings from this study highlight that stigma in healthcare settings functions as a barrier to HIV treatment and care, particularly for women who face multiple layers of marginalization. Among both Black and non-Black WWH who use drugs, stigma is strongly associated with suboptimal adherence and viral nonsuppression. Although overall stigma levels were lower among Black and non-Black WWH who do not use drugs, and there were no substantial differences between these groups in this regard, they are not immune to stigma's effects and may still experience other forms of discrimination or structural disadvantage that impact their HIV care. In contrast, stigma was markedly higher among women who use drugs, regardless of race, underscoring substance use as a critical axis of marginalization within healthcare settings.

To optimize viral suppression across these subgroups, efforts must prioritize decreasing stigma through cultural norms shifts and reducing structural barriers to HIV care. Additionally, programs that integrate HIV care, harm reduction services that address drug use, offer mental health support and economic empowerment strategies, especially for WWH who use drugs, may have a profound impact on improving HIV outcomes and advancing intersectional equity among US WWH. Taken together, this research may help inform the development of future interventions and guide resource allocation by highlighting the subgroups of women most at risk of poor HIV treatment and care outcomes and offering insights to inform targeted care strategies.

## Notes

***Acknowledgments.*** The authors gratefully acknowledge the contributions of the study participants and dedication of the staff at the MACS/WIHS Combined Cohort Study Data (MWCCS) sites.

***Author contributions.*** J. P. J. and T. B. N. conceptualized the study. J. P. J. designed the analysis and wrote the manuscript. N. E. L. analyzed the data. J. S. and P. C. T. contributed to the study design and revised the manuscript. C. D.-R., M. O. J., A. S., B. T., A. A., M.-C. K., G. D., M. F.-M., S. K., A. S., A. T., and D. J. W. provided edits. All authors reviewed and approved the final version of the manuscript.

***Data availability.*** Access to individual-level data from the MWCCS may be obtained upon review and approval of a MWCCS concept sheet. Links and instructions for online concept sheet submission are on the study website.

***Financial support.*** Data in this manuscript were collected by the Women's Interagency HIV Study (WIHS), now the MWCCS. The contents of this publication are solely the responsibility of the authors and do not represent the official views of the National Institutes of Health. MWCCS (Principal Investigators): Atlanta (Clinical Research Site [CRS]) (Ighovwerha Ofotokun, Anandi Sheth, and Gina Wingood), U01-HL146241; Baltimore CRS (Todd Brown and Joseph Margolick), U01-HL146201; Bronx CRS (Kathryn Anastos, David Hanna, and Anjali Sharma), U01-HL146204; Brooklyn CRS (Deborah Gustafson and Tracey Wilson), U01-HL146202; Data Analysis and Coordination Center (Gypsyamber D'Souza, Stephen Gange, and Elizabeth Topper), U01-HL146193; Chicago–Cook County CRS (Mardge Cohen, Audrey French, and Ryan Ross), U01-HL146245; Chicago-Northwestern CRS (Steven Wolinsky, Frank Palella, and Valentina Stosor), U01-HL146240; Northern California CRS (Bradley Aouizerat, Jennifer Price, and Phyllis Tien), U01-HL146242; Los Angeles CRS (Roger Detels and Matthew Mimiaga), U01-HL146333; Metropolitan Washington CRS (Seble Kassaye and Daniel Merenstein), U01-HL146205; Miami CRS (Maria Alcaide, Margaret Fischl, and Deborah Jones), U01-HL146203; Pittsburgh CRS (Jeremy Martinson and Charles Rinaldo), U01-HL146208; University of Alabama at Birmingham (UAB)–MS CRS (Mirjam-Colette Kempf, James B. Brock, Emily Levitan, and Deborah Konkle-Parker), U01-HL146192; and University of North Carolina (UNC) CRS (M. Bradley Drummond and Michelle Floris-Moore), U01-HL146194. The MWCCS is funded primarily by the National Heart, Lung, and Blood Institute, with additional co-funding from the Eunice Kennedy Shriver National Institute of Child Health and Human Development, National Institute on Aging, National Institute of Dental and Craniofacial Research, National Institute of Allergy and Infectious Diseases, National Institute of Neurological Disorders and Stroke, National Institute of Mental Health, National Institute on Drug Abuse, National Institute of Nursing Research, National Cancer Institute, National Institute on Alcohol Abuse and Alcoholism, National Institute on Deafness and Other Communication Disorders, National Institute of Diabetes and Digestive and Kidney Diseases, and National Institute on Minority Health and Health Disparities, and in coordination and alignment with the research priorities of the National Institutes of Health, Office of AIDS Research. MWCCS data collection is also supported by UL1-TR000004 (University of California, San Francisco Clinical and Translational Science Award [CTSA]), UL1-TR003098 (Johns Hopkins University Institute for Clinical and Translational Research), UL1-TR001881 (University of California, Los Angeles Clinical and Translational Science Institute), P30-AI-050409 (Atlanta Center for AIDS Research [CFAR]), P30-AI-073961 (Miami CFAR), P30-AI-050410 (UNC CFAR), P30-AI-027767 (UAB CFAR), P30-MH-116867 (Miami Center for HIV and Research in Mental Health), UL1-TR001409 (DC CTSA), KL2-TR001432 (DC CTSA), and TL1-TR001431 (DC CTSA). J. P. J. is supported by a Career Development Award from the National Institute on Drug Abuse (award number 5K01DA056306).
